# Head and Neck Examinations Among Patients Presenting to HRSA‐Funded Health Centers in the United States

**DOI:** 10.1111/jphd.70001

**Published:** 2025-06-23

**Authors:** Leah I. Leinbach

**Affiliations:** ^1^ National Institute of Dental & Craniofacial Research, National Institutes of Health Bethesda Maryland USA

**Keywords:** community health centers, head and neck neoplasms, oral examinations, oral health, prevention

## Abstract

**Objective:**

Health centers funded by the Health Resources and Services Administration (HRSA) are a safety net for people who may not be able to access care elsewhere. Patients eligible for care at these facilities share some of the same risk factors for developing head and neck cancer. The objective of this study is to examine the prevalence of head and neck cancer examinations among patients of HRSA‐funded health centers.

**Methods:**

This is an analysis of the cross‐sectional 2022 Health Center Patient Survey (HCPS). Self‐reported data from this survey of patients of health centers regarding receipt of head and neck examinations is summarized, with results stratified by sociodemographic, behavioral, and health‐related factors.

**Results:**

Four thousand four hundred and fourteen unweighted patients (20,693,940 weighted) participated in the HCPS, 69.5% of whom answered questions about a head and neck exam. Of these, 9.9% of patients reported a history of a head and neck exam (HNE). Patients from minoritized racial/ethnic groups were less likely to report an HNE compared to White, non‐Hispanic patients. Patients with Medicare, Medicaid, and lower incomes were also less likely to report an HNE compared to patients with private insurance and incomes above $50,000 per year. A dental exam anywhere within the last year was associated with a report of an HNE.

**Conclusions:**

Disparities in reported HNEs were observed by patient race/ethnicity, medical payor, income, and recency of dental visit among patients of US health centers. Health centers may be uniquely positioned to explore and generate evidence regarding HNEs that could inform changes in practice.

## 1 Introduction

Head and neck cancers (HNCs) are a heterogeneous group of cancers involving the oral cavity, pharynx, larynx, paranasal sinuses, nasal cavity, and salivary glands [1]. Risk factors for HNC include smoking, heavy alcohol use, and persistent infection with human papillomavirus (HPV), particularly HPV‐16 and 18 [1, 2]. Approximately 4% of all cancers diagnosed in the United States are HNCs [3], with the incidence of HPV‐related oropharyngeal cancers projected to increase until at least 2045 [4]. In 2024, the incidence of HNCs in the United States is estimated to be 58,000 cases, with about 12,000 deaths, both figures heavily skewed toward men [5].

Consistent with other cancer types, earlier detection of HNCs improves prognosis, requires less morbid treatments, increases survival rate, and leads to better post‐treatment quality‐of‐life and lower cost of care [1, 2, 6]. Roughly one quarter of HNCs are diagnosed with localized spread [5], suggesting an opportunity to increase the proportion of cancers identified when prognosis is improved and morbidities reduced [7]. HNCs may be detected through visual and tactile examination of the oral cavity and surrounding structures, including the dorsal, ventral, and lateral surfaces of the tongue and floor of mouth, as well as palpation of the neck [3]. Examination of the neck is especially relevant for the capture of HPV‐related oropharyngeal cancers, the initial presentation of which is often a solitary neck mass [2]. Because of the many advantages resulting from early detection, increasing the proportion of HNCs detected at the earliest stage is one of the Healthy People 2030 Oral Health goals [8].

Reported rates of head and neck exams are low across all patient populations, with the lowest rates among individuals who are low‐income [9]. For example, Semprini et al. found that only 5.6% of lower‐income NHANES 2017 respondents under 65 reported an oral cancer examination [9]. Patients with HNCs are more likely to belong to minoritized racial or ethnic groups, be male, have lower income, rely on public insurance, and have lower educational attainment and poorer overall health compared to patients with other cancers [6]. Patients who are Black have an overall lower incidence of HNC but higher mortality, longer delays in care, and lower rates of guideline‐adherent treatment compared to White patients and patients from other minoritized groups [10]. Lower median income and not having private medical insurance have also been associated with higher 90‐day mortality [11].

HRSA‐funded health centers are a safety net for many low‐income people in the United States, providing lower‐cost, sliding‐scale care for people who may otherwise not be able to access treatment [12]. These facilities deliver a range of services such as medical checkups, cancer screenings, oral health care services, and case management to upwards of 30 million patients annually [12, 13]. As such, health centers are uniquely positioned to close the demographic gaps in early detection of pre‐malignant and malignant lesions of the head and neck, including among lower‐income populations. This study aims to evaluate the prevalence of head and neck cancer exams (HNEs) among patients at HRSA‐funded health centers and to identify key predictors of such exams among this vulnerable patient population.

## 2 Methods

### 2.1 Study Design and Data Source

This is an analysis of the cross‐sectional 2022 Health Center Patient Survey (HCPS). The HCPS is an in‐person interview survey of over 4400 patients from up to 300 HRSA‐funded health centers in the United States [14]. Weighting is applied to yield a nationally representative sample of patients of health centers.

### 2.2 Study Population and Primary Outcome

The study population was adult patients of HRSA‐funded health centers who answered both of two questions related to intra‐ and extra‐oral head and neck exams in the 2022 HCPS: (1) “Have you ever had an exam for oral cancer in which the doctor or dentist pulls on your tongue, sometimes with gauze wrapped around it, and feels under the tongue and inside the cheeks?” and (2) “Have you ever had an exam for oral cancer in which the doctor or dentist feels your neck?” [14]. The primary outcome of this study was a reported history of an HNE, specifically a visual and tactile assessment of the oral cavity and neck. A history of HNE was defined as a ‘yes’ to both of the above questions.

### 2.3 Sociodemographic, Behavioral and Health‐Related Factors

The HCPS also gathers information regarding sociodemographic characteristics including patient age, sex, race/ethnicity, and marital status, and behavioral characteristics such as prior year smoking status (combustion cigarettes). Health‐related factors include patient's self‐reported overall health status ranging from excellent to poor, medical payor type (public, private, or uninsured), presence or absence of dental benefits, and dental exam or medical exam anywhere within the last year. Information about neighborhood characteristics (i.e., rurality) and annual income were also collected.

### 2.4 Statistical Methods

All data were weighted per HCPS guidelines to estimates generalized to the US health center population unless otherwise specified. Characteristics of the study population were reported as frequencies and percentages, with differences between populations with and without reported HNEs using chi‐square testing. Multivariable logistic regression was used to identify predictors of an HNE among demographic factors, overall health status, insurance, income, rurality, history of dental exam, and history of medical exam, accounting for complex survey design. A *p* < 0.05 was considered significant. All analyses were conducted using SAS 9.4 (Cary, NC) survey procedures accounting for complex study design and weighting. Analysis was performed in August 2024. This study was exempted from IRB review as it used publicly available data. Findings are reported per STROBE guidelines for cross‐sectional studies.

## 3 Results

### 3.1 Study Population

In total, 4414 patients participated in the survey, representing a weighted total of 29,693,940 patients. Of these, 3867 (20,896,544 weighted) answered the question about an oral cancer exam and 3856 (20,749,416 weighted) answered the question about a neck exam. 69.5% of patients (20,636,105 weighted) answered both of these questions. Of these, 9.9% (2,050,183 weighted) reported a head and neck exam, while 92.1% (18,585,922 weighted) did not report an exam. All respondents to this question were adults aged 18 or older.

### 3.2 Sociodemographic, Behavioral, & Health Characteristics

The study sample was predominantly female (63.0%), non‐Hispanic White (43.5%), between the ages of 18 and 44 (50.6%), and not married (56.4%). 30.5% reported an annual income of less than $15,000, and 35.4% between $15,000 and $35,000 (Table 1), verifying that at least two‐thirds of patients of health centers reported annual incomes below the 2024 federal poverty level for a family of four [15]. Most patients had either Medicaid (38.8%) as the primary payor or were uninsured/self‐pay (26.5%), with 69.0% reporting a dental benefits plan. 32.7% of patients were from rural areas, and 48.7% were from communities identified as urban or suburban. 40.4% rated their overall health as fair or poor, and 22.4% reported current cigarette smoking. Most (72.2%) had a routine medical checkup with a primary care provider within the last year, but only 52.3% had a dental exam within the last year.

**TABLE 1:** Characteristics of study population, Health Center Patient Survey (2022).

Characteristic	Frequency, *n* = 3834	Weighted frequency^a^, *n* = 20,636,105	Weighted, %	SE
Age				
18–44	1354	10,443,282	50.6	2.3
45–64	1849	7,094,684	34.4	2.1
65–74	510	2,471,152	12.0	1.4
75+	121	626,986	3.0	0.8
Sex				
Male	1194	7,637,355	37.0	2.3
Female	2640	12,998,749	63.0	2.3
Race/ethnicity				
Hispanic	1507	4,953,068	24.0	3.8
Non‐Hispanic White	973	8,933,384	43.5	4.6
Non‐Hispanic Black	825	3,571,410	17.3	3.6
Other	529	3,178,242	15.4	2.8
Marital status				
Married or domestic partner	1455	9,000,742	43.6	1.9
Not married	2379	11,635,363	56.4	1.9
Smoking status				
Current smoker	867	4,623,383	22.4	2.1
Non‐smoker	2959	15,995,495	77.6	2.1
Overall health status				
Excellent	210	1,194,987	5.80	0.8
Very good	515	3,665,408	17.8	1.5
Good	1349	7,400,224	35.9	1.6
Fair or poor	1754	8,325,023	40.4	2.3
Medical payor				
Private	565	4,202,964	20.4	2.2
Medicare	864	2,957,330	14.4	1.5
Medicaid, other public	1870	8,001,490	38.8	3.1
Uninsured/self‐pay	535	5,474,321	26.5	4.1
Dental benefits				
Yes	2400	12,836,164	69.0	2.5
No	1232	5,768,015	31.0	2.5
Income				
< 15 k	1520	6,313,653	30.5	1.8
15–35 k	1307	7,291,479	35.4	1.9
35–50 k	490	3,189,570	15.4	1.7
50 k+	517	3,841,402	18.6	2.0
Rurality				
Rural	955	6,750,500	32.7	3.7
Suburban	671	3,867,351	18.7	2.0
Urban	1396	6,190,453	30.0	3.1
Other or unknown	812	3,827,801	18.6	1.6
Dental exam w/in last year				
At health center				
Yes	707	3,270,194	15.9	2.1
No	3095	17,292,868	84.1	2.1
Anywhere				
Yes	1976	10,762,454	52.3	1.8
No	1829	9,809,411	47.7	1.8
Medical exam w/in last year				
At health center				
Yes	2695	15,544,310	75.3	1.6
No	1139	5,091,794	24.7	1.6
Anywhere				
Yes	2687	14,904,393	72.2	1.9
No	1147	5,731,712	27.8	1.9

Abbreviation: SE, standard error of the weighted %.

^a^ Weighting applied per HCPS guidelines.

### 3.3 Factors Associated With a Head and Neck Exam

**FIGURE 1:**
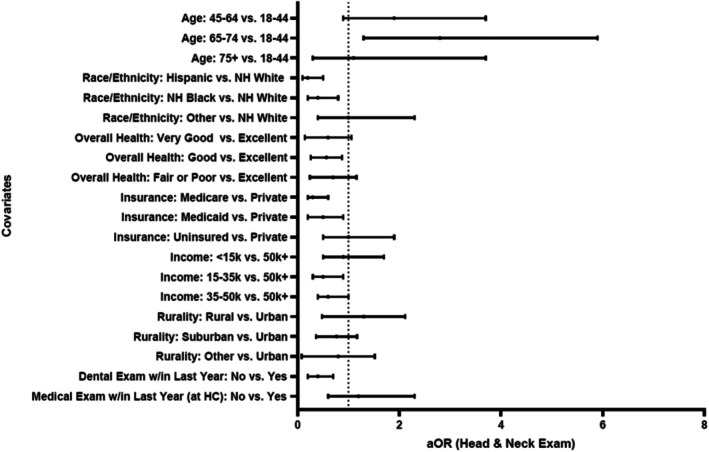
Factors associated with a head and neck exam, adjusted, HCPS (2022). HCPS, Health Center Patient Survey, weighting applied per HCPS guidelines, model statistic = 0.71.

Unadjusted analysis showed that patient age, race/ethnicity, overall health status, medical insurance status, income, rurality, and having a dental exam in the last year were each associated with having a head and neck exam (Table 2). Patient race/ethnicity, medical payor, income, and recent dental exam remained statistically significant after weighted adjusted analysis (Figure 1, Table S1). Hispanic and non‐Hispanic Black patients were less likely to report an HNE compared to non‐Hispanic White patients, with odds ratios of 0.2 (95% CI: 0.1–0.5, *p* = < 0.0001) and 0.4 (95% CI: 0.2–0.8, *p* = < 0.0001), respectively. Regarding payor type, patients with Medicare were least likely to report an HNE compared to patients with private insurance (OR: 0.3, 95% CI: 0.2–0.6, *p* = 0.0002), with the likelihood also much lower among patients with Medicaid (OR: 0.5, 95% CI: 0.2–0.9, *p* = 0.0002). Patients with annual incomes between $15 and $35 k were the least likely to report a head and neck exam compared to those with incomes $50 k or higher (OR: 0.5, 95% CI: 0.3–0.9, *p* = 0.03). Patients who did not have a dental exam in the last year were less likely to report a head and neck exam compared to those who saw a dentist in the last year (OR: 0.4, 95% CI: 0.2–0.7, *p* = 0.0009).

**TABLE 2:** Factors associated with a head and neck exam, unadjusted, HCPS (2022).

Characteristic, *n* = 20,636,105 (100)	*n* (weighted %)^a^		SE	*p* (*χ* ^2^)
	(+) HNE, *n* = 2,050,183 (9.9)	(−) HNE, *n* = 18,585,922 (90.1)		
Age				
18–44	741,230 (7.1)	9,702,052 (92.9)	1.5	0.004^a^
45–64	839,997 (11.9)	6,254,688 (88.1)	1.8	
65–74	417,079 (16.9)	2,054,073 (83.1)	3.6	
75+	51,877 (8.3)	575,110 (91.7)	3.7	
Sex				
Male	814,068 (10.7)	6,823,287 (89.3)	2.0	0.61
Female	1,236,114 (9.5)	11,762,635 (90.4)	1.4	
Race/ethnicity				
Hispanic	184,799 (3.7)	4,768,270 (96.3)	1.1	0.0001^a^
Non‐Hispanic White	1,300,241 (14.6)	7,633,143 (85.4)	2.2	
Non‐Hispanic Black	217,314 (6.1)	3,354,097 (93.9)	1.6	
Other	347,829 (10.9)	2,830,413 (89.1)	3.5	
Marital status				
Married or domestic partner	966,670 (10.7)	8,034,073 (89.3)	2.3	0.60
Not married	1,083,513 (9.3)	10,551,850 (90.7)	1.5	
Smoking status				
Current smoker	432,506 (9.4)	4,190,877 (90.6)	1.9	0.75
Non‐smoker	1,612,946 (10.1)	14,382,549 (89.9)	1.5	
Overall health status				
Excellent	207,543 (17.3)	987,443 (82.6)	6.0	0.006^a^
Very good	412,428 (11.3)	3,252,979 (88.7)	2.6	
Good	582,165 (7.9)	6,818,058 (92.1)	1.9	
Fair or poor	841,435 (10.1)	7,483,588 (90.0)	1.4	
Medical payor				
Private	825,214 (19.6)	3,377,750 (80.3)	4.0	< 0.0001^a^
Medicare	238,459 (8.1)	2,718,870 (91.9)	1.7	
Medicaid, other public	489,079 (6.1)	7,512,411 (93.9)	1.5	
Uninsured/self‐pay	497,431 (9.1)	4,976,890 (90.9)	1.9	
Dental benefits				
Yes	1,307,064 (10.2)	11,529,100 (89.8)	1.5	0.75
No	542,090 (9.4)	5,225,925 (90.6)	2.3	
Income				
< 15 k	603,983 (9.6)	5,709,669 (90.4)	1.8	0.0002^a^
15–35 k	434,488 (6.0)	6,856,991 (94.0)	1.2	
35–50 k	290,197 (9.1)	2,899,373 (90.9)	2.6	
50 k+	721,514 (18.8)	3,119,889 (81.2)	3.9	
Rurality				
Rural	997,295 (14.8)	5,753,205 (85.2)	2.3	0.007^a^
Suburban	306,299 (7.9)	3,561,051 (92.1)	2.2	
Urban	536,482 (8.7)	5,653,971 (91.3)	1.9	
Other or unknown	210,106 (5.4)	3,617,695 (94.5)	1.8	
Dental exam w/in last year				
At health center				
Yes	354,712 (10.8)	2,915,482 (89.2)	2.7	0.70
No	1,692,923 (9.8)	15,599,944 (90.2)	1.3	
Anywhere				
Yes	1,446,283 (13.4)	9,316,171 (86.6)	2.1	0.001
No	601,352 (6.1)	9,208,059 (93.9)	1.3	
Medical exam w/in last year				
At health center				
Yes	1,552,037 (10.0)	13,992,273 (90.0)	1.5	0.94
No	498,145 (9.8)	4,593,649 (90.2)	2.4	
Anywhere				
Yes	1,691,398 (11.3)	13,212,994 (88.7)	1.6	0.08
No	358,784 (6.3)	5,372,928 (93.7)	1.9	

Abbreviations: HCPS, Health Center Patient Survey; SE, standard error of weighted %.

^a^ Weighting applied per HCPS guidelines.

## 4 Discussion

This study describes factors associated with a history of head and neck exam among patients of HRSA‐funded health centers in 2022. The overall proportion of patients surveyed who reported a head and neck exam was low, under 10% of the sample population, consistent with trends observed in national‐level studies [9]. Previous research has also shown that screenings for other cancers (i.e., breast, cervical, and colorectal) are also lower among health center populations compared to national estimates [16]. Differences in reported HNEs were observed based on patient race/ethnicity, medical payor, income, and having a dental exam in the last year. Reports of an HNE were higher among middle and older adult age groups (45 years and older), in alignment with the groups most likely to be diagnosed with head and neck cancer [17], although differences were not statistically significant. Smoking and male sex, known risk factors for HNC, were not associated with a head and neck cancer exam in this analysis.

Patients identified as non‐Hispanic White, with private medical insurance and a comparatively higher annual income (above $50,000) were the most likely to have reported a head and neck exam. This study population had a higher proportion of patients identified as Hispanic (43.5%) compared to 2020 US Census estimates (19.5%), with lower comparative proportions of patients identified as non‐Hispanic White [18]. Prior research shows that patients from certain minoritized groups are less likely to be engaged in conversation about risk factors for head and neck cancers by dentists [19], more likely to be diagnosed at later stages of disease [17], and less likely to have guideline‐adherent care [20]. Further, patients with medical coverage through Medicare were least likely to report an HNE, followed by patients with Medicaid and those who were uninsured. Currently, Medicare does not include coverage for routine HNEs by oral health care providers, and the financial barriers to accessing dental care remain high [6], although theoretically these barriers should be reduced by the payment structure of HRSA‐funded health centers. The lower rates of HNEs observed among patients with Medicare and Medicaid, and those with lower income (i.e., working poor), are consistent with the disparities outlined above.

Slightly over half of the study participants reported a dental exam in the last year, most of which were performed by providers outside of the health center (84.1%). Patients who reported a dental exam within the last year were 60% more likely to report having a head and neck examination, suggesting that dentists may be primarily responsible for performing this type of exam, particularly in settings outside of a health center. This may be due to varying levels of oral health services and differing practice models between private settings and health centers. In addition, these findings suggest that patients may be unaware that an HNE occurred, or the exam may have not included an assessment of the oral cavity/tongue and neck. This underscores the need for an HNE at all new patient and follow‐up visits, in accordance with recommendations by the American Dental Association [21], and patient education to ensure that patients are aware of their purpose and importance.

Further, these findings highlight a potential opportunity to leverage medical visits to increase HNEs, particularly given that medical visits were more common than dental visits in this population. Prior research suggests that a targeted approach to earlier identification of HNCs among the highest‐risk groups may be the most cost‐effective [22]. Health centers may be uniquely positioned to contribute to the body of evidence through well‐considered pilot programs, similar to those used for other types of cancers [23]. Such pilot programs could leverage resources such as shared electronic health records and co‐location of multiple different types of providers to inform approaches applicable in both health centers and other settings.

This study has several limitations. First, this study is cross‐sectional in nature and therefore only provides information about the current state at one point in time. Second, data is subject to the recall and response biases inherent in self‐reported survey data. Third, the HCPS does not include information about the timing or situation of a head and neck exam, making it impossible to know when or by what provider type the HNEs were performed. Fourth, information about other risk factors (i.e., HPV status, alcohol status) were either not available in this dataset or had a high proportion of missing data. Finally, data from this survey may reflect anomalies related to the disruptions of the COVID‐19 pandemic, including telemedicine visits, the extent of which cannot be determined. Telemedicine contacts are not amenable to HNEs, which require visualization deep into the oral cavity and careful palpation of the neck and the floor of the mouth. Additional research into post‐COVID HCPS data may shed light on the effect of remote compared to in‐person medical screening on the prevalence of HNEs. Despite those limitations, findings from this study provide insight into the current state of HNEs generalizable to US health centers.

In summary, this study demonstrates disparities in reported HNEs by patient race/ethnicity, medical payor, income, and recency of dental visit among patients of US health centers using data from the 2022 Health Center Patient Survey. Health centers may be uniquely positioned to explore and generate evidence regarding HNEs that may change practice in both health centers and in other settings. Targeted studies that further examine the patient, provider, and health system‐level perspectives could be of benefit.

## Conflicts of Interest

The author declares no conflicts of interest.

## Supporting information


**Table S1.** Factors associated with a head and neck exam, adjusted, HCPS (2022).

## Data Availability

The data that support the findings of this study are available in HCPS dashboard at https://bphc.hrsa.gov/data‐reporting/health‐patient‐survey. These data were derived from the following resources available in the public domain: HRSA Health Center Patient Survey, https://bphc.hrsa.gov/data‐reporting/health‐center‐patient‐survey.
