# Supplementary material for: Head and Neck Examinations Among Patients Presenting to HRSA‐Funded Health Centers in the United States
Source: J Public Health Dent. 2025 Jun 23;85(4):380–7. doi: 10.1111/jphd.70001 (PMC12264812; doi:10.1111/jphd.70001)
Supplement: Supplementary file 1 — Table S1. Factors associated with a head and neck exam, adjusted, HCPS (2022). [file JPHD-85-380-s001.docx]

**Supplementary Table 1: Factors Associated with a Head and Neck Exam, Adjusted, HCPS (2022)**

| Characteristic | aOR | Lower CI | Upper CI | p value |
| --- | --- | --- | --- | --- |
| *Age* |  |  |  |  |
| 18-44 (Ref) | 1.0 | 1.0 | 1.0 | 0.08 |
| 45-64 | 1.8 | 0.9 | 3.5 |  |
| 65-74 | 2.7 | 1.2 | 5.4 |  |
| 75+ | 1.0 | 0.3 | 3.7 |  |
| *Race / Ethnicity* |  |  |  |  |
| Hispanic | 0.2 | 0.1 | 0.5 | <.0001* |
| Non-Hispanic White (Ref) | 1.0 | 1.0 | 1.0 |  |
| Non-Hispanic Black | 0.4 | 0.2 | 0.8 |  |
| Other | 1.0 | 0.4 | 2.7 |  |
| *Overall Health Status* |  |  |  |  |
| Excellent | 1.0 | 1.0 | 1.0 | 0.12 |
| Very Good | 0.5 | 0.2 | 1.1 |  |
| Good | 0.5 | 0.3 | 0.9 |  |
| Fair or Poor | 0.6 | 0.3 | 1.2 |  |
| *Medical Payor* |  |  |  |  |
| Private (Ref) | 1.0 | 1.0 | 1.0 | 0.0002* |
| Medicare | 0.3 | 0.2 | 0.6 |  |
| Medicaid, Public (non-Medicaid) | 0.5 | 0.2 | 0.9 |  |
| Uninsured / Self-Pay | 1.0 | 0.5 | 1.9 |  |
| *Income* |  |  |  |  |
| <15k | 0.9 | 0.4 | 1.9 | 0.03* |
| 15-35k | 0.5 | 0.3 | 0.9 |  |
| 35-50k | 0.6 | 0.3 | 1.0 |  |
| 50k+ (Ref) | 1.0 | 1.0 | 1.0 |  |
| *Rurality* |  |  |  |  |
| Rural | 1.1 | 0.6 | 2.2 | 0.27 |
| Suburban | 0.7 | 0.4 | 1.2 |  |
| Urban (Ref) | 1.0 | 1.0 | 1.0 |  |
| Other or Unknown | 0.6 | 0.2 | 1.6 |  |
| *Dental Exam Anywhere w/in Last Year* |  |  |  |  |
| Yes (Ref) | 1.0 | 1.0 | 1.0 | 0.003* |
| No | 0.4 | 0.2 | 0.7 |  |
| *Medical Exam w/in Last Year at HC* |  |  |  |  |
| Yes (Ref) | 1.0 | 1.0 | 1.0 | 0.53 |
| No | 1.2 | 0.6 | 2.4 |  |

HCPS = Health Center Patient Survey; HC=Health Center

Weighting applied per HCPS guidelines

Model Statistic = 0.71
